# Human influence on brown trout juvenile body size during metapopulation expansion

**DOI:** 10.1098/rsbl.2021.0366

**Published:** 2021-10-27

**Authors:** L. Aulus-Giacosa, F. Guéraud, P. Gaudin, M. Buoro, J. C. Aymes, J. Labonne, M. Vignon

**Affiliations:** Universite de Pau et des Pays de l'Adour, E2S UPPA, INRAE, ECOBIOP, Saint-Pée-sur-Nivelle ou Anglet, France

**Keywords:** dispersal, density dependence, brown trout, subantarctic, invasion biology

## Abstract

Change in body size can be driven by social (density) and non-social (environmental and spatial variation) factors. In expanding metapopulations, spatial sorting by means of dispersal on the expansion front can further drive the evolution of body size. However, human intervention can dramatically affect these founder effects. Using long-term monitoring of the colonization of the remote Kerguelen islands by brown trout, a facultative anadromous salmonid, we analyse body size variation in 32 naturally founded and 10 human-introduced populations over 57 years. In naturally founded populations, we find that spatial sorting promotes slow positive changes in body size on the expansion front, then that body size decreases as populations get older and local density increases. This pattern is, however, completely different in human-introduced populations, where body size remains constant or even increases as populations get older. The present findings confirm that changes in body size can be affected by metapopulation expansion, but that human influence, even in very remote environments, can fully alter this process.

## 1. Introduction

Body size is a fundamental trait known to be linked with metabolic rate, physiology, life history (reproduction, competition, survival, dispersal) and consequently fitness [1]. The direction of changes in body size is controlled by many within-population factors like increase in competition [2], but also local environment effects on metabolism and selection and related selective pressures [3]. Body size, therefore, seems to evolve adaptively through genetic selection and phenotypic plasticity with regard to both social environment and biotic or abiotic environment [2,4–6]. Dispersal between populations, however, can also have an effect on changes in body size [7,8]. Indeed, if dispersers represent a non-random sample of the origin population in terms of body size wise [7,9], and provided that body size at age is inheritable and they manage to reproduce locally, they may have an effect on the structure of the recipient population.

Both processes within and between populations driving body size evolution are likely to be strongly impacted by the current pace and magnitude of global change [10,11]. Many species undergo shifting expansion ranges, simultaneously encountering novel environments due to climate change. Humans also transported and introduced species intentionally or not in new environments where they might first settle, then eventually invade [12]. These situations present an adequate context to study the pace and direction of body size changes, since they enable the study of sharp density gradients from the core to the moving edge of the distribution area. A classic case of such expansion is found in newly founded metapopulations [13,14], wherein habitat is not continuous. This pattern implies the colonization of new habitat patches to found new local populations, thus expanding further the metapopulation, with dispersal events between patches [15].

At the core of the metapopulation, as populations get older, the body size is generally expected to decrease as local density increases, triggering competition over resources [16], except if the cost of evolving bigger body size is compensated by fitness gains [17]. But on the expanding range, because establishing new natural populations involves dispersal, if dispersers represent a particular genotype and phenotype of the core populations regarding body size [7,9], then spatial sorting may occur [18,19]. Those individuals will be over-represented in newly founded populations [20], where they will undergo reduced competition due to low densities. Such bias could be further promoted along the expanding range if body size at age (growth) is heritable [21], thereby producing observable clines in body size at age.

Other mechanisms, however, may tamper with these general expectations: local environment may trigger phenotypic plasticity in growth, producing different body sizes and affecting dispersal propensity [3,22]. Yet in many cases, these mechanisms are further shaped by human intervention: numbers and stages of propagules introduced, or genetic origins, habitat degradation. Plus, these introduced populations will not be influenced by spatial sorting. The evolution of body size is thus likely to be controlled by both ecological mechanisms and multiple human influences [23].

**Figure 1.**
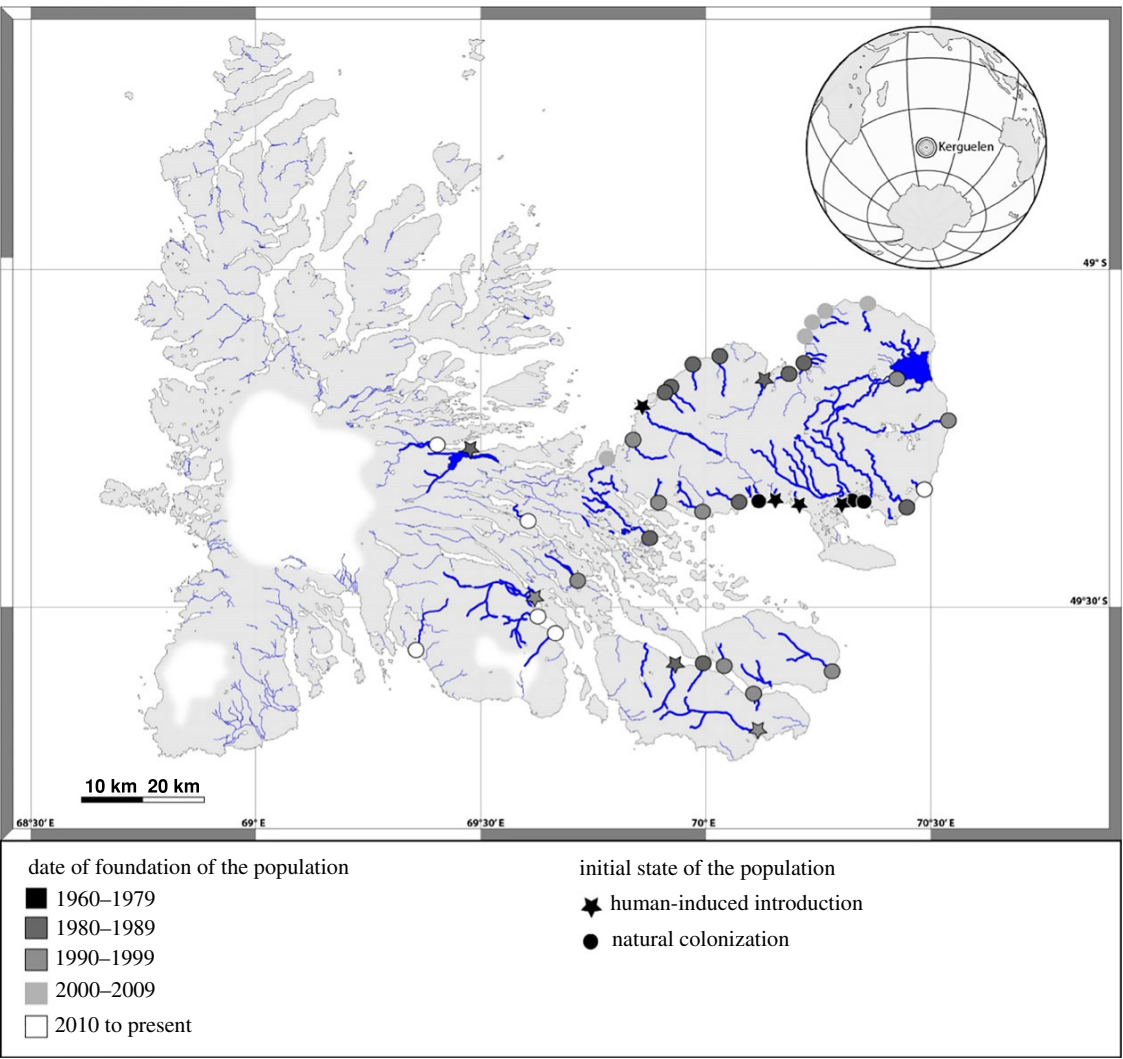
Maps of the Kerguelen Islands showing the colonized rivers included in the study.

To investigate and disentangle the above hypotheses, we considered a facultative anadromous species, the brown trout (*Salmo trutta* L.), where part of individuals are resident (entire life cycle in fresh waters) and part of individuals migrate at sea (anadromy) out of the reproductive window [24]. Brown trout is a philopatric species, reproducing in their natal rivers and forming distinct populations, except for some anadromous who disperse eventually. Because body size at age is related to migration propensity [25,26] while anadromy and length-at-migration are partly heritable in many salmonids species [3,21], one can expect spatial sorting arising in brown trout as well. Moreover, anadromous females have higher body size compared to resident ones, and larger body size is positively correlated to greater fecundity and egg size [3]. This means that the population founded by anadromous females are expected to be composed of bigger body-sized and more numerous juveniles. This species has been introduced all over the world for more than a century [27], and is considered to be the most invasive vertebrate [28,29]. The subantarctic Kerguelen islands is no exception, giving access to the founding of a metapopulation monitored from the very beginning, currently spanning more than 42 populations and still expanding [30] (figure 1). Resident fish were used to found stocked populations (*n* = 10) while some populations have been naturally founded (*n* = 32) via dispersal [31]. Benefiting from a 57-years long-term monitoring (1962–2019), we investigate how juvenile body size is influenced by phenotypic sorting during natural metapopulation expansion (founder effect, measured by the founding date of new populations relative to the metapopulation founding date), how juvenile size changes as the population grows (increasing local density approached by the age since founding of each population) and how human influence changes in juvenile body size pattern (stocked versus naturally founded population) (figure 1).

## 2. Material and methods

We investigated specifically juvenile body size during the second year of growth, when fish are still living in freshwater and before sexual maturation occurs, so growth reflects the local environment. Based on our long-term data collection [32] in which part of the samples are aged [33], we used Neural Network Learning (knn function of class R package, v. 7.3–14, see electronic supplementary material, file S1) to detect and retrieve a selection of 21 639 one-year-old individuals. Their precise body size (fork length, mm) and Julian day of capture in the year were noted, as well as their sampling location (river). These 21 639 fish were distributed over 42 populations and 53 years (from 1967 to 2019, electronic supplementary material, file S1) (figure 1).

To investigate a change in body size, we used a linear mixed-effects model. We tested the effect of two fixed continuous factors (population age and metapopulation age, age being the number of years since founding, respectively) and one categorical factor (colonization status: natural or introduced) and their interactions on the relationship between the logarithm of body size and the day of capture. Population age was taken as a proxy of density increase [34], reflecting local level for competition [35]. Metapopulation age in interaction with population age was used to capture the effect of spatial sorting (for naturally colonized populations) on the expanding range of the colonized area. We included the sampling locations (rivers) as random effects on both the intercept and the slope of the relationship between body size and capture date. This approach allowed the local environment effects that can affect growth—often through phenotypic plasticity—to be removed from the general replicated trends that we sought to uncover at the metapopulation scale. The full model integrating all parameters to estimate was written as follows:

$$\log\;(BS_{i,j})∼N(\mu_{i,j},\;\sigma^{2})$$

$$\begin{array}{rl} \mu_{i,j}= & \;\theta_{0}+\theta_{1}\times S_{j}+\theta_{2}\times D_{i}+\theta_{3}\times M_{i}+\theta_{4}\times P_{i,j}+\theta_{5}\times M_{i}\\ & \;\times P_{i,j}+\theta_{6}\times D_{i}\times M_{i}+\theta_{7}\times D_{i}\times P_{i,j}+\theta_{8}\times D_{i}\times M_{i}\\ & \;\times P_{i,j}+\theta_{9}\times D_{i}\times S_{j}+\theta_{10}\times M_{i}\times S_{j}+\theta_{11}\times P_{i,j}\times S_{j}\\ & \;+\theta_{12}\times M_{i}\times P_{i,j}\times S_{j}+\theta_{13}\times D_{i}\times M_{i}\times S_{j}+\theta_{14}\times D_{i}\\ & \;\times P_{i,j}\times S_{j}+\theta_{15}\times D_{i}\times M_{i}\times P_{i,j}\times S_{j}+\alpha_{j}+\beta_{j}\times D_{i} \end{array}$$

Wherein *BS_i,j_* is the body size of individual *i* in river *j*, *D* is the Julian day of capture, *M* is the metapopulation age, *P* is the population age, *S* is the colonization status, *θ_k_* are the fixed parameters to estimate, *α_j_* and *β_j_* are the river random effect for the intercept and slope of the relationship, respectively. We used the package lme4 in *R* software to estimate the model's parameters (electronic supplementary material, file S2), with a stepwise approach to fit the full model and all nested models of interest. In order to assess the statistical importance of each fixed factor in the model, the Bayesian information criterion (BIC) [36] was used to compare the different models.

## 3. Results

**Figure 2.**
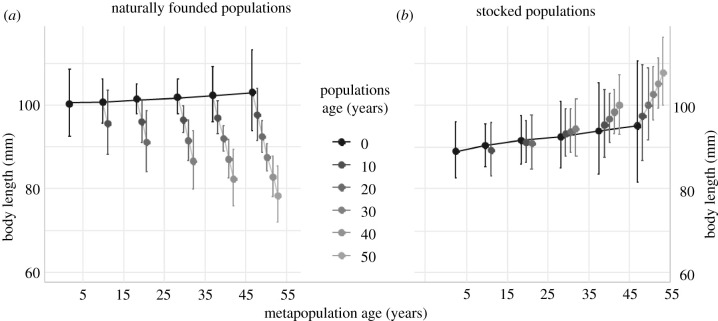
Average body size in millimetres (dots) with 2.5–97.5% CI (vertical segments) predicted (using fixed effects) at the mean capture date (226th day of the second year) by the best model, as a function of metapopulation age and population ages for (*a*) naturally founded populations and (*b*) stocked populations. The black lines between dots indicate changes of body size in newly founded populations along the expanding range, the grey lines indicate changes of body size when populations get older.

The comparison of models using the BIC approach indicates that the most complex model provides the best predictive model for the data (electronic supplementary material, file S2). This implies that the ages of metapopulation and populations, respectively, as well as the status of river colonization (stocked or naturally colonized) all significantly and interactively influence variation in body size (figure 2), whereas local environment effects were included in the random effects estimates (electronic supplementary material, file S2). As expected under spatial sorting, body size changed along the expanding range: in new naturally founded populations, body size increased slowly toward greater values (3 mm over 50 years). In these populations, as time since founding increased, body size rapidly decreased (20 mm over 50 years), as anticipated under increased competition for resource. In stocked populations, body size was overall much smaller, and increased slowly in recently stocked populations. But as time since population founding increased, body size increased, as opposed to naturally founded populations.

## 4. Discussion

Looking at changes in juvenile body size over a half-century (10–15 generations), our analysis aimed at revealing large scale patterns driven by metapopulation expansion. Although we found evidence that change in body size was correlated to local increase in density and spatial sorting (related to dispersal as anticipated), we also showed a clear contrast between naturally founded populations and human stocked populations.

We found that juvenile body size is influenced by phenotypic sorting during natural expansion (founder effect), with slow-paced changes towards bigger body sizes. The increase in body size through spatial sorting is consistent with the available literature, where migrants are generally fast-growing individuals [25,26]. It also matches with predictions of body size—dispersal reaction norms in metapopulations where dispersal cost is reduced and environmental stochasticity is substantial [8,37], a likely scenario for the brown trout in Kerguelen. In turn, such evolution may have contributed to the observed extension of the dispersal kernel [30,38]. Likewise, r-selection on the expanding range should drive a positive evolution in body size [7], notably because body size in fish is indeed positively correlated to the age at maturation in most cases [39–42] and strongly positively correlated to fecundity [21,43]. However, average changes in juvenile body size are small. This may be partly due to the imperfect pattern of spatial expansion: some rivers near the core of the metapopulation were colonized only recently, due to their small size that reduces their attractiveness [30]. This may have decreased the effect of spatial sorting in our analysis, since the geographical proximity between such recently colonized patches with the core of the metapopulation will facilitate gene flow between populations founded at very different dates, also presenting contrasted densities [44]. Although spatial sorting occurs when body size at age is heritable, we cannot in the present analytical framework assess the relative contributions of pure genetic factors versus parental and epigenetic effects.

In stocked rivers, juvenile body size also increases in the most recently introduced rivers, but this pattern here cannot be related to spatial sorting. Interestingly, body size measured soon after the introduction was always lower than in newly naturally colonized rivers, possibly indicating strong non-adaptive founder effects related to introduction conditions or already strong competition induced by propagule pressure. The various genetic origins, numbers and life stages of propagules used to stock these rivers [31] may also have profound evolutionary consequences, in shaping the genetic and phenotypic foundation of these human introduced populations, notably the relationship between density and growth rate of individuals [45], whereas dispersal drives the founder effects in naturally colonized systems. The average juvenile body size within naturally founded versus stocked populations could also arise from the fact that naturally founded populations were founded by anadromous females with larger eggs (inducing larger juveniles, [46]) compared to stocked populations founded by resident females with smaller eggs. The increase in juvenile body size in stocked populations could be related to slower increase in density within stocked populations in relation to the size of fish, therefore, experiencing better early juvenile growth.

Juvenile body size in naturally founded populations decreases as the population grows (increasing local density) as expected under a competition for resource hypothesis [16]. The speed, extent and replicability of body size changes was remarkable in that case, seemingly overriding any potential effect of spatial sorting. Plasticity in growth in relation to density is abundantly documented among fish and salmonids [47,48], but our results provide a new temporal appraisal of the importance of density dependence in unstable populations. As expected under phenotypic plasticity, a large amount of local variation in growth unrelated to density dependence was also found, captured by the random effects in the model. Part of this variation might, however, be the result of natural selection: subantarctic islands present poor trophic freshwater ecosystems, and trout in Kerguelen feed on and metabolize carbohydrates during early stages [49], to adapt to the oligotrophic conditions in freshwater.

More strikingly, we found no evidence of a decrease in body size as stocked populations aged, whereas density steadily increased [34]. We assume that as the proportion of anadromous females increases over time in the stocked populations, birth size increases accordingly. Whereas in naturally founded population anadromous contribution should decrease over time, stocked populations are expected to become more anadromous through time (starting at 0% with the resident fry). These life-history transitions might go a long way in predicting and explaining the observed changes in fry size (natural getting smaller, stocked getting bigger). Changes in the juvenile density and/or stream productivity will modify these patterns until the resident—anadromous ratio settles onto each river's life history ‘adaptive peak’. Moreover, it is likely that the rivers where fish were introduced are not a random subset of the ecological spectrum available: these systems are usually large, with easy access to sea, and generally not far from human presence. They also bear the ecological footprints of human presence: they can, therefore, undergo selection on body size through fishing, community assemblages are modified through various species introductions, affecting ecosystem dynamics and productivity [50].

To disentangle relative parts of phenotypic plasticity and genetic evolution in the observed patterns of body size evolution, large scale investigation of population genetics in relation to ecosystem-level processes seems a mandatory avenue [51]. This is especially pertinent as other factors might rapidly affect these dynamics, such as climate change, the effects of which are particularly strong in subantarctic and Antarctic ecosystems [52–54]. These forecasted changes will interact strongly with salmonids, as they are pioneer species that benefit from their sea migration to effect rapid nutrient transfer between ecosystems, improving river productivity [55], hence changing growth opportunities for future generations.

In conclusion, while our analysis generally concurs to support the idea that density gradients, and to a lesser extent spatial sorting, are two important drivers of rapid changes in juvenile body size in expanding metapopulations [7], the impact of human footprints was manifest, even in this remote location where human presence is strongly limited [56].

## Data accessibility

We hereby state that the data for the present study are fully accessible for reviewers as well as readers. They can be downloaded at the following address: https://doi.org/10.15454/7BCTVN. Description of the data can be found along with the data in the online repository. Description of the variables are in the main.txt file. The data are provided in the electronic supplementary material [57].
